# Experimental dataset on the residual performance of fiber-reinforced cementitious composite subjected to high temperature

**DOI:** 10.1016/j.dib.2022.108745

**Published:** 2022-11-12

**Authors:** Paulo Roberto Ribeiro Soares Junior, Priscila de Souza Maciel, Elaine Carballo Siqueira Correa, Augusto Cesar da Silva Bezerra

**Affiliations:** a Department of Materials Engineering, Federal Center for Technological Education of Minas Gerais (CEFET-MG), Avenida Amazonas, 5253, Belo Horizonte, MG 30421-169, Brasil; b Department of Transport Engineering, Federal Center for Technological Education of Minas Gerais (CEFET-MG), Avenida Amazonas, 5253, Belo Horizonte, MG 30421-169, Brasil

**Keywords:** Fiber-reinforced cementitious composite, High temperature, Flexural behavior, Pull-out response, Fiber-matrix analysis

## Abstract

The present dataset refers to the research article entitled “A multiscale investigation on the performance improvement of fiber-reinforced cementitious composites after exposure to high temperatures” [1]. Supplementary data on raw materials characterization, temperature recording, mass loss, water absorption, compressive strength, flexural behavior, pull-out response, fiber-matrix interface, and surface, microstructure and hardness of fibers are presented here. The continuous matrix was produced from cementitious grout containing Portland cement, sand, silica fume, superplasticizer, and water. The heating was carried out in an electric oven up to 260 °C. The bending tests was performed for fiber-reinforced cementitious composite (FRCC) with steel fiber contents of 1%, 3%, and 5% by volume, and for non-fibrous matrix. The pull-out test was performed using single fiber embedded in the matrix. The water absorption and axial compression tests was performed for non-fibrous matrix. The fiber-matrix analysis was performed from polished sections of fibers embedded in cementitious matrix. The fiber analysis was performed from steel fibers. The data refer to the residual properties after heating and slow cooling or to the reference condition without heating. The data can help in understanding residual performance of FRCC after exposure to high temperatures and may be useful for developing resilient building materials.

**Specifications Table**

**Table:** 

Subject	Civil and Structural Engineering
Specific subject area	Construction materials
Type of data	Table Image Graph Figure
How the data were acquired	The data presented in this article were obtained from the following laboratory tests: - Temperature measurement: K-type thermocouples - 4-point bending test: UTM* EMiC, 23DL (300 kN load cell) - Axial compression test: UTM EMiC, 23DL (300 kN load cell) - Single fiber pull-out test: UTM EMiC, 23DL (20 kN load cell) - Nanoindentation test: Microdurometer Shimadzu, HMV - Image analysis: Optical microscopy Kontrol, IM713 *UTM – Universal Test Machine
Data format	Raw Analysed Filtered
Parameters for data collection	The age of 7 days was used for all tests. The high temperature submission was set at 260 °C. For FRCC, fiber contents of 1%, 3% and 5% by volume were used.
Description of data collection	The data were collected from the test of specimens, produced through the methodology of slurry infiltrated fibers for bending, cementitious grout (non-fibrous) for compression and water absorption, and single fibers for pull-out. The fiber characterization was performed from embedded fibers (microstructure and hardness) and individual fibers (surface). The fiber-matrix interface was evaluated from fibers immersed in the cement matrix. Half of the specimens were exposed to high temperature and tested in a residual condition, after slow cooling. The other half was tested without heating.
Data source location	Institution: Federal Centre for Technological Education of Minas Gerais City/Town/Region: Belo Horizonte, Minas Gerais Country: Brazil
Data accessibility	With this article and online Mendeley Data: *Raw data on fiber-reinforced cementitious composite subjected to high temperature* URL: https://data.mendeley.com/datasets/hc3bct2byc doi: 10.17632/hc3bct2byc.1 *Analyzed data on fiber-reinforced cementitious composite subjected to high temperature* URL: https://data.mendeley.com/datasets/tb6bkc3g5m doi: https://doi.org/10.17632/tb6bkc3g5m.1
Related research article	[1] P.R.R. Soares Junior, P. de S. Maciel, E.C.S. Corrêa, A.C. da S. Bezerra, A multiscale investigation on the performance improvement of fiber-reinforced cementitious composites after exposure to high temperatures, Cem Concr Compos. 133 (2022) 104,657. doi: https://doi.org/10.1016/j.cemconcomp.2022.104657

## Value of the Data

• These data are important for predicting and understanding the behavior of fiber reinforced cementitious composites (FRCCs) subjected to high temperatures.
• The data was obtained from experimental tests at different levels of analysis (multiscale – macro, meso and microstructural). This approach provides a valuable dataset for developing resilient materials.
• Engineers, designers, and researchers can benefit from this data, particularly from the residual performance improvement after high temperatures.
• Researchers working on the development of materials optimized for severe temperatures would recognize the value of this data.
• This dataset can be useful in the design of fire-resistant structures and development of heat-cured precast structures.
• The data can be useful to calibrate, verify, and validate numerical and analytical models in predicting the behavior of FRCCs under fire conditions.

## 1 Data Description

The data presented in this dataset refers to the supplementary data from the article entitled “A multiscale investigation on the performance improvement of fiber-reinforced cementitious composites after exposure to high temperatures” . This section has been divided into subsections related to: Raw materials characterization (1.1); Ambient temperature record (1.2); Mass loss (1.3); Water absorption (1.4); Compressive strength (1.5); Flexural behavior (1.6); Pull-out response (1.7); Fiber microstructure (1.8); Fiber hardness (1.9); Fiber surface (1.10); and Fiber-matrix interface (1.11). The no-heat condition was defined as AM and the data obtained after submission to high temperature of HT. The raw and analyzed data are available in Mendeley Data (see Ref [2,3].).

### 1.1 Raw materials characterization

Table 1, Table 2, Table 3, Table 4, Table 5 show the detailed characterization of the raw materials used. All data were obtained from manufacturers and suppliers.

**Table 1:** Physical-chemical characterization of Portland cement.

Tests	Methodology	Unit	Result	Requirement
Insoluble residue	ABNT NM 15/12	%	0.57	≤ 1.0
Loss of ignition	ABNT NM 18/12	%	3.75	≤ 4.5
Magnesium oxide	ABNT NM 21/12	%	1.48	≤ 6.5
Sulfur oxide	ABNT NM 16/12	%	2.73	≤ 4.5
Carbon dioxide	ABNT NM 20/12	%	2.61	≤ 3.0
Surface area (Blaine)	ABNT NM 76/98	cm^2^/g	4507	≥ 3000
Sieve residue #200	ABNT NBR 11579/91	%	0.06	≤ 6.0
Sieve residue #325	ABNT NBR 9202/85	%	0.87	–
Normal consistency water	ABNT NM 43/03	%	30.4	–
Setting start	ABNT NM 65/03	min	142	≥ 60
Setting end	ABNT NM 65/03	min	191	≤ 600
Expandability	ABNT NBR 11582/91	mm	0.00	≤ 5.0

Source: Brennand cements.

**Table 2:** Characteristics of steel fiber.

Characteristic	Steel fiber
Type / nomenclature by manufacturer	FF3
Material	Low carbon steel
Manufacturing method	Cold forming (drawing)
Section shape	Circular
Length (mm)	50
Diameter (mm)	0.75
l/d (form factor)	67
Tensile strength (limit of resistance)	1200 MPa

Source: Maccaferri.

**Table 3:** Chemical composition of silica fume.

Chemical analysis,%	Silica fume
Silicon dioxide (SiO_2_)	91.04
Aluminum oxide (Al_2_O_3_)	0.10
Iron oxide (Fe_2_O_3_)	0.70
Calcium oxide (CaO)	1.10
Magnesium oxide (MgO)	1.50
Sodium oxide (Na_2_O)	0.39
Potassium oxide (k_2_o)	4.40
Sulfur trioxide (so_3_)	0.16
Loss of ignition	0.61

Source: Tecnosil.

**Table 4:** Properties of superplasticizer.

Property	Result
Aspect	Viscous liquid
Color	Sienna
Homogeneity	Homogeneous
Specific mass	1.09 g/cm^3^
Solid residue content	47.8%
pH	3.1
Chloride content	≤ 0.15%

Source: Tecnosil.

**Table 5:** Properties of water.

Property	Result
Color	Colourless
Turbidity	Clear
Temperature	Ambient, around 25 °C
pH	7.83
Chloride	1.09 mg/L
Fluoride	0.8 mg/L

Source: Copasa.

Table 1 shows the physical-chemical characteristics of Portland cement, as well as tests performed and methodology adopted, with 0.57% (<1.0) of insoluble residue, 3.75% (<4.5) of loss of ignition, 1.48% (<6.5) of magnesium oxide, 2.73% (<4.5) of sulfur oxide, 2.61 (<3.0) of carbon dioxide, 4.507 cm2/g (>3000) of surface area (Blaine), 0.06% (<6.0) of sieve residue #200, 0.87% of residue on the #323 sieve, 30.4% of normal consistency water, 142 min of start setting (> 60), 191 min of end setting (<600) and 0.00 mm of hot expandability (<5.0).

Table 2 shows the characteristics of steel fibers. The fiber nomenclature was designated as (FF3) according to the manufacturer, the material is low carbon steel, the manufacturing method was cold-forming (drawing), the section shape is circular with length (l) of 50 mm and diameter (d) 0.75 mm, form factor equals to 67 (l/d) and tensile strength (limit of strength) of 1200 MPa.

Table 3 shows the chemical composition of silica fume, which had 91.04% silicon dioxide, 0.10% aluminum oxide, 0.70% iron oxide, 1.10% calcium oxide, 1.50% magnesium oxide, 0.39% sodium oxide, 4.40% potassium oxide, 0.16% sulfur trioxide and 0.61% loss of ignition.

Table 4 shows the properties of the superplasticizer, which has a homogeneous viscous liquid appearance, siena color, 47.8% solid residue content, 3.1 pH and ≤0.15% chloride content.

Table 5 shows the properties of the water used in the mixture of the fluid mortar, which had a colorless and clear appearance, at room temperature around 25 °C, 1.09 mg/L of chloride and 0.8 mg/L of fluoride.

### 1.2 Ambient temperature record

**Fig. 1:**
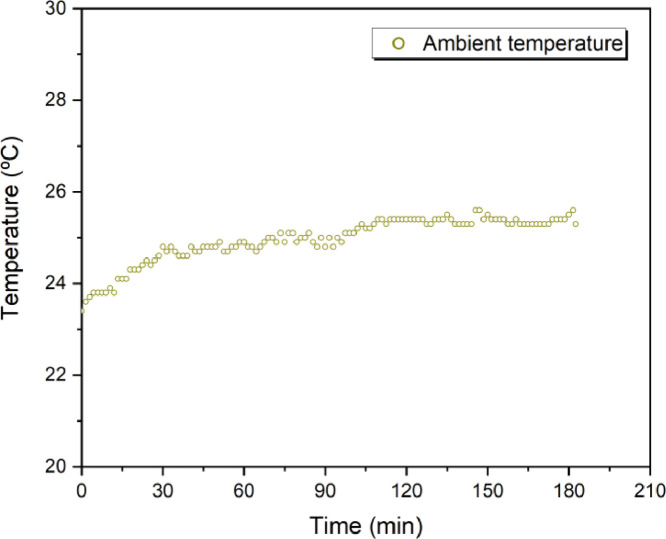
Record of ambient temperature.

Fig. 1 shows the record of ambient temperature during submission of specimens to high temperature. The data are in the range between 23.4 °C and 25.6 °C, over approximately 120 min of measurement.

### 1.3 Mass loss

Tables 6 and 7 show the raw data for mass loss and relative mass loss, both due to high temperature submission. The average mass loss of the six specimens was 19.17 g after drying in an oven and 7.50 g after heat treatment. The total mass loss was 26.66 g. The average relative mass loss was 4.88% after oven drying, 2.02% after heat treatment and 6.90% in total.

**Table 6:** Data of mass loss.

Sample	ML - OV (g)	ML - HT (g)	ML - Total
1	18.85	7.84	26.69
2	19.97	7.68	27.65
3	18.93	8.13	27.06
4	19.56	8.32	27.88
5	18.81	7.02	25.83
6	18.87	5.99	24.86
Average	19.17	7.50	26.66
Standard deviation	0.44	0.78	1.04
Coefficient of variation	0.023	0.10	0.039

Note: ML – mass loss; OV- oven; HT – heat treatment.

**Table 7:** Data of relative mass loss.

Sample	ML - OV (%)	ML - HT (%)	ML - Total (%)
1	4.72	2.06	6.78
2	5.21	2.11	7.32
3	4.74	2.14	6.88
4	5.06	2.27	7.33
5	4.83	1.89	6.73
6	4.73	1.57	6.31
Average	4.88	2.01	6.90
Standard deviation	0.20	0.24	0.38
Coefficient of variation	0.04	0.12	0.05

Note: ML – mass loss; OV- oven; HT – heat treatment.

### 1.4 Water absorption

Tables 8, 9 and 10 show the data from the water absorption test.

**Table 8:** Saturated, dry, and submerged mass, before and after heat treatment.

Sample	AM			HT		
	M_ssd_ (g)	M_dry_ (g)	M_sub_ (g)	M_ssd_ (g)	M_dry_ (g)	M_sub_ (g)
1	399.25	380.40	209.24	393.21	372.56	202.85
2	383.17	363.20	191.67	375.11	355.52	183.34
3	398.70	379.77	207.86	393.53	371.64	203.05
4	385.89	366.33	194.96	378.50	358.01	188.68
5	389.02	370.21	198.46	382.10	363.19	192.52
6	398.38	379.51	207.73	391.06	373.52	200.94

Note: M_dry_ - dry mass; M_ssd_ - saturated mass dry-surface; M_sub_ - submerged-saturated mass.

**Table 9:** Data of water absorption, dry bulk density, saturated bulk density and porosity for the unheated condition.

Sample	WA (%)	DBD (g/cm^3^)	SBD (g/cm^3^)	PR (%)
1	4.96	2.00	2.10	9.25
2	5.50	1.90	2.00	9.44
3	4.98	1.99	2.09	9.02
4	5.34	1.92	2.02	9.29
5	5.08	1.94	2.04	8.98
6	4.97	1.99	2.09	9.00
Average	5.14	1.96	2.06	9.13
Standard deviation	0.2272	0.0437	0.0418	0.1915
Coefficient of variation	0.0442	0.0224	0.0203	0.0210

Note: WA - water absorption; DBD - dry bulk density; SBD - saturated bulk density; PR - porosity.

**Table 10:** Data of water absorption, dry bulk density, saturated bulk density and porosity after high temperature.

Sample	WA (%)	DBD (g/cm^3^)	SBD (g/cm^3^)	PR (%)
1	5.54	1.96	2.07	9.78
2	5.51	1.85	1.96	9.26
3	5.89	1.95	2.07	10.30
4	5.72	1.89	1.99	9.74
5	5.21	1.92	2.02	9.06
6	4.70	1.96	2.06	8.44
Average	5.43	1.92	2.03	9.44
Standard deviation	0.4256	0.0444	0.0451	0.6508
Coefficient of variation	0.0784	0.0231	0.0222	0.0690

Note: WA - water absorption; DBD - dry bulk density; SBD - saturated bulk density; PR - porosity.

Table 8 shows the weighing data of the six specimens used, in the following ways: dry mass in oven or after HT (M_dry_), saturated mass dry-surface (M_ssd_) and submerged-saturated mass (M_sub_). For the AM condition, the M_dry_ data are comprised between 363.20 g and 380.40 g, M_ssd_ between 383.17 g and 399.25 g, and M_sub_ between 191.67 g and 209.24 g. For the HT condition, the M_dry_ data are comprised between 355.52 g and 373.53 g, M_ssd_ 375.11 g and 393.53 g, and M_sub_ between 183.34 g and 202.85 g.

Table 9 shows the water absorption (WA), dry bulk density (DBD), saturated bulk density (SBD) and porosity (PR) data for the unheated condition. The WA data are between 4.96% and 5.50%, average of 5.14%. DBD data are between 1.90 g/cm^3^ and 2.00 g/cm^3^, average of 1.96 g/cm^3^. The SBD data are between 2.00 g/cm^3^ and 2.10 g/cm^3^, average of 2.06 g/cm^3^. PR data are between 8.98% and 9.44%, average of 9.13%.

Table 10 shows the WA, DBD, SBD and PR data after high temperature submission. The WA data are between 4.70% and 5.89%, average of 5.43%. DBD data are between 1.85 g/cm^3^ and 1.96 g/cm^3^, average of 1.92 g/cm^3^. The SBD data are between 1.96 g/cm^3^ and 2.07 g/cm^3^, average of 2.03 g/cm^3^. PR data are between 8.44% and 10.30%, average of 9.44%.

### 1.5 Compressive strength

Table 11 shows the compressive strength data of the cementitious matrix (non-fibrous). For the AM condition, the strength was between 67.05 MPa and 83.87 MPa, average of 70.53 MPa and standard deviation of 3.4119 MPa. For HT, the strength was between 85.51 MPa and 89.66 MPa, mean of 88.07 MPa and standard deviation of 2,2385 MPa.

**Table 11:** Compressive strength of cementitious matrix.

Condition	Sample	Strength	Average	Standard deviation
AM	SP1	67.05	70.53	3.4119
	SP2	70.66		
	SP3	73.87		
HT	SP1T	85.51	88.07	2.2385
	SP2T	89.04		
	SP3T	89.66		

### 1.6 Flexural behavior

**Fig. 2:**
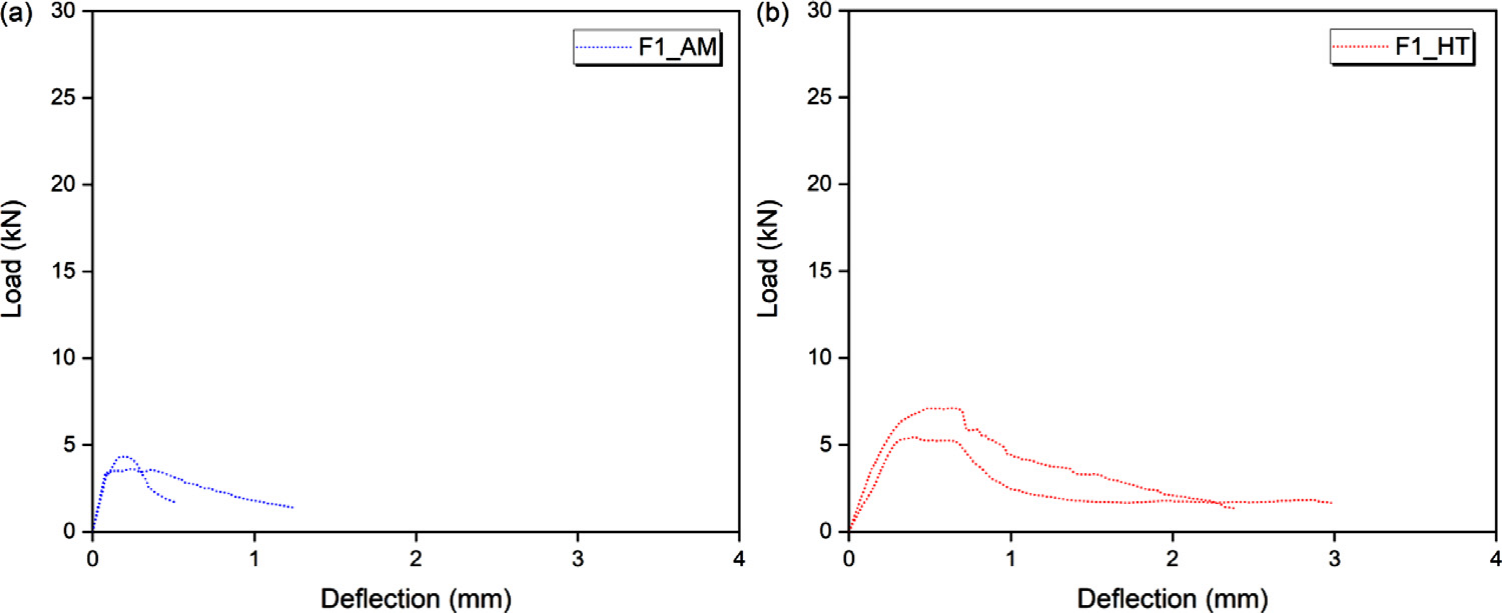
Flexural behavior of (a) F1AM and (b) F1HT.

**Fig. 3:**
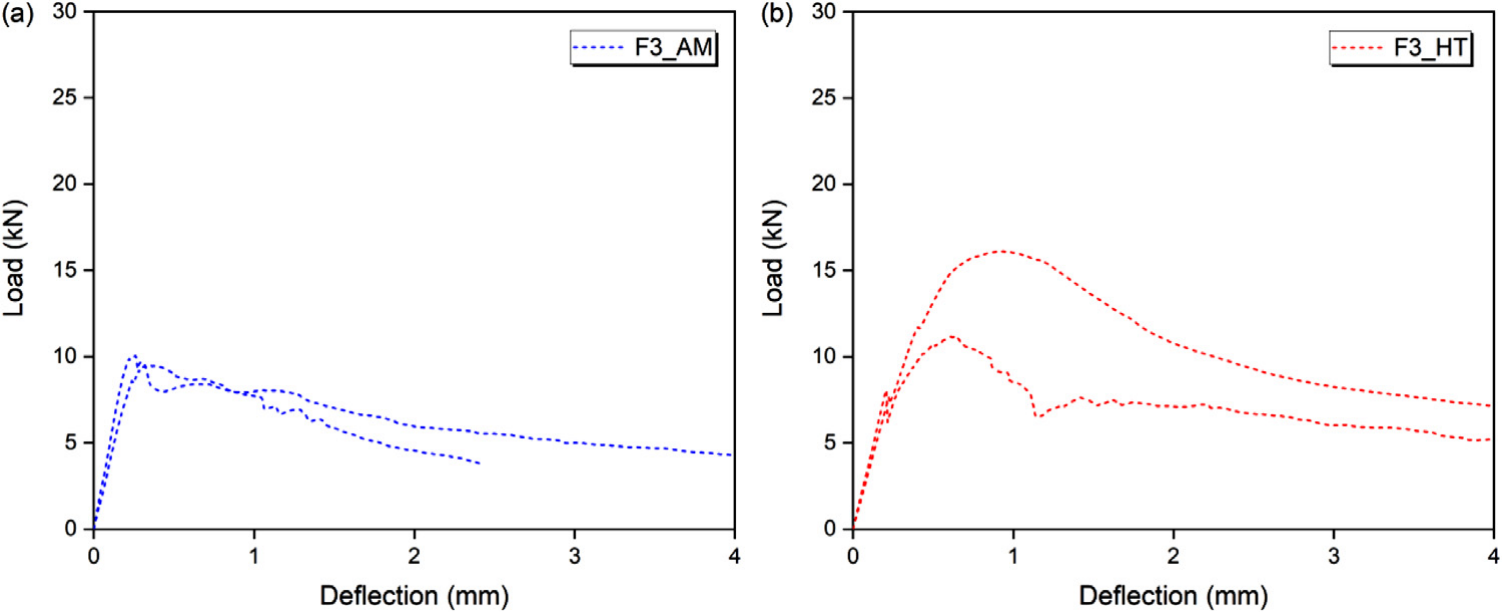
Flexural behavior of (a) F3AM and (b) F3HT.

**Fig. 4:**
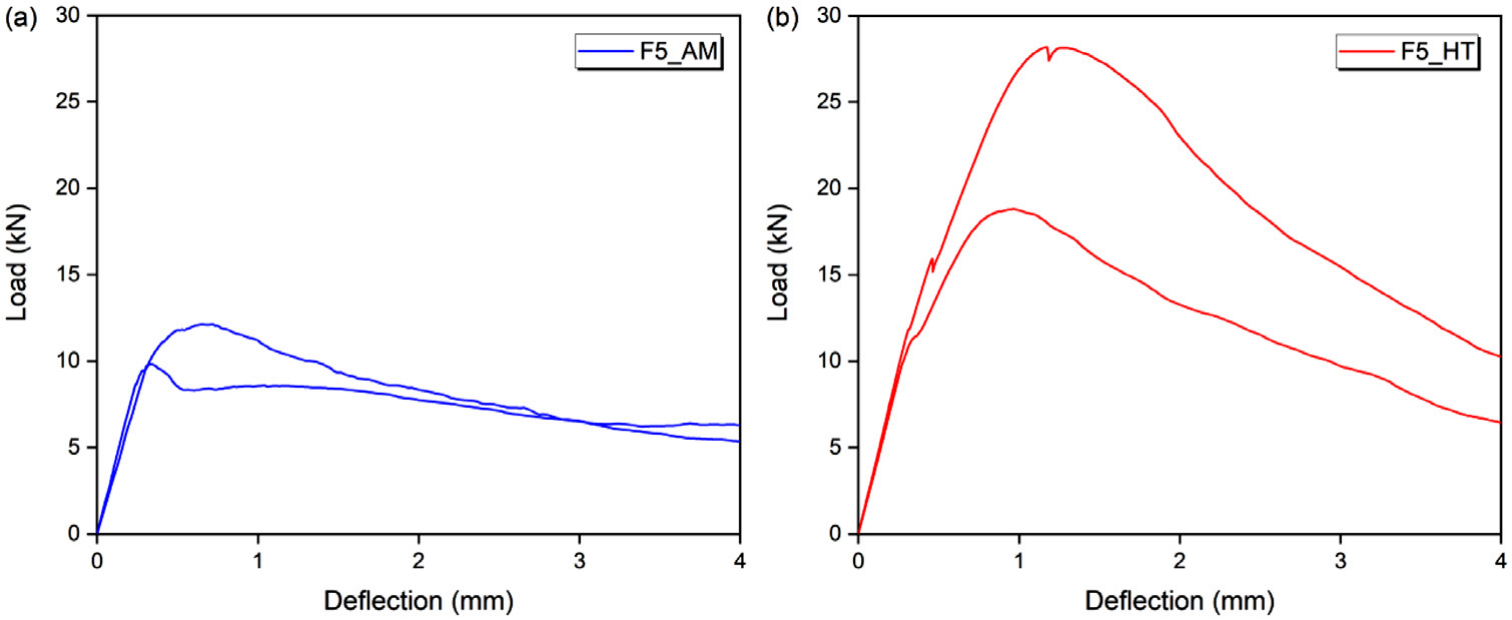
Flexural behavior of (a) F5AM and (b) F5HT.

Figs. 2, 3 and 4 show the flexural behavior of FRCC without heating (AM) and after heat treatment (HT). The fiber content is designated as “F”, that is, 1%, 3% and 5% of fibers correspond to F1, F3 and F5. Data from two specimens is shown for each condition.

Table 12, Table 13, Table 14, Table 15 show the bending behavior parameters, in terms of load, strength, deflection and toughness, for three specimens in each condition. The respective average values and standard deviations are also shown.

Table 12 shows the load (P_max_), strength (σ_P,max_) and deflection (δ_P,max_) corresponding to the maximum load or peak load. The maximum load remained between 1511 N (non-fibrous matrix, without heating) to 28,205 N (FRCC with 5% fiber, after heat treatment). The strength started at 3.12 MPa and went up to 58.17 MPa, while the deflection was from 0.049 mm to 1401 mm, for the same composites and conditions (matrix-AM and F5HT).

Table 13 shows the toughness before the peak, at the point corresponding to 0.9 of the maximum load (T_0.9P,bp_). The toughness of the cementitious (non-fibrous) matrix was negligible. Toughness started at 125 N.mm (FRCC with 1% fibers, without heating) and went up to 12,984 N.mm (FRCC with 5% fibers, after heat treatment).

Table 14 shows the toughness at the point corresponding to the peak load (T_P,pc_). The toughness of the cementitious (non-fibrous) matrix was negligible. Toughness started at 418 N.mm (FRCC with 1% fibers, without heating) and went up to 23,499 N.mm (FRCC with 5% fibers, after heat treatment).

Table 15 shows the toughness after the peak, at the points corresponding to 0.9, 0.7 and 0.5 of the maximum load (T_0.9P,pp_, T_0.7P,pp_ and T_0.5P,pp_). For T_0.9P,pp_ the toughness was between 637 N.mm and 37,226 N.mm; for T_0.7P,pp_ between 1086 N.mm and 49,876 N.mm; and for T_0.5P,pp_ between 1276 N.mm and 67,297 N.mm; all in relation to F1AM and F5HT, respectively.

**Table 12:** Load, strength, and deflection at peak.

		P_max_ (N)			σ_P,max_ (MPa)			δ_P,max_ (mm)		
Sample		P_max_	Md	Sd	σ_P,max_	Md	Sd	δ_P,max_	Md	Sd
Matrix-AM	SP1	1814	1696	162	3.74	3.50	0.333	0.069	0.059	0.010
	SP2	1764			3.64			0.057		
	SP3	1511			3.12			0.049		
F1AM	SP1	4248	4062	394	8.76	8.38	0.82	0.157	0.190	0.039
	SP2	3609			7.44			0.233		
	SP3	4329			8.93			0.180		
F3AM	SP1	9662	9726	294	19.93	20.06	0.61	0.338	0.317	0.049
	SP2	9469			19.53			0.352		
	SP3	10047			20.72			0.260		
F5AM	SP1	12115	11473	1421	24.99	23.66	2.93	0.643	0.424	0.190
	SP2	9844			20.30			0.328		
	SP3	12460			25.70			0.301		
Matrix-HT	SP1	2312	2160	158	4.77	4.46	0.326	0.095	0.104	0.008
	SP2	2170			4.48			0.110		
	SP3	1997			4.12			0.106		
F1HT	SP1	7117	6113	887	14.68	12.61	1.83	0.640	0.434	0.191
	SP2	5434			11.21			0.399		
	SP3	5789			11.94			0.262		
F3HT	SP1	14356	13876	2509	29.61	28.62	5.18	0.610	0.710	0.176
	SP2	16110			33.23			0.913		
	SP3	11162			23.02			0.607		
F5HT	SP1	24525	23849	4730	50.58	49.19	9.75	1.401	1.180	0.217
	SP2	28205			58.17			1.174		
	SP3	18817			38.81			0.967		

Notes: P_max_ = P – maximum load or peak load; σ_P,max_ – strength; δ_P,max_ – deflection at peak load; Md – average; Sd – standard deviation.

**Table 13:** Toughness before the peak load at 0.9P

			T_0.9P,bp_ (N.mm)		
Sample		0.9P,bp	T_0.9P,bp_	Md	Sd
Matrix-AM	SP1	–	–	–	–
	SP2	–	–		
	SP3	–	–		
F1AM	SP1	3823	217	216	90
	SP2	3248	125		
	SP3	3896	305		
F3AM	SP1	8696	965	1016	164
	SP2	8522	1199		
	SP3	9042	884		
F5AM	SP1	10904	2351	1610	646
	SP2	8860	1162		
	SP3	11214	1317		
Matrix-HT	SP1	–	–	–	–
	SP2	–	–		
	SP3	–	–		
F1HT	SP1	6405	1228	829	347
	SP2	4891	657		
	SP3	5210	602		
F3HT	SP1	12920	3731	3765	1098
	SP2	14499	4880		
	SP3	10046	2685		
F5HT	SP1	22073	11984	10526	3429
	SP2	25385	12984		
	SP3	16935	6609		

Notes: P_max_ = P – maximum load or peak load; 0.9P,bp – point before the peak, which the load is 0.9 of the maximum load; T_0.9P,bp_ – toughness at point 0.9P,bp; Md – average; Sd – standard deviation.

**Table 14:** Toughness at peak load.

			T_pc_ (N.mm)		
Sample		P,pc	T_pc_	Md	Sd
Matrix-AM	SP1	–	–	–	–
	SP2	–	–		
	SP3	–	–		
F1AM	SP1	4248	418	534	131
	SP2	3609	676		
	SP3	4329	507		
F3AM	SP1	9662	2157	1955	324
	SP2	9469	2127		
	SP3	10047	1582		
F5AM	SP1	12115	5324	3180	1870
	SP2	9844	1888		
	SP3	12460	2329		
Matrix-HT	SP1	–	–	–	–
	SP2	–	–		
	SP3	–	–		
F1HT	SP1	7117	3386	1892	1307
	SP2	5434	1328		
	SP3	5789	962		
F3HT	SP1	14356	5205	6633	2986
	SP2	16110	10064		
	SP3	11162	4629		
F5HT	SP1	24525	23499	18670	5880
	SP2	28205	20389		
	SP3	18817	12123		

Notes: P_max_ = P – maximum load or peak load; P,bp – point at peak load; T_0.9P,bp_ – toughness at point P,bp; Md – average; Sd – standard deviation.

**Table 15:** Toughness post-peak at 0.9P, 0.7P and 0.5P

			T_0.9P,pp_ (N.mm)				T_0.7P,pp_				T_0.5P,pp_		
Sample		0.9P,pp	T_0,9P,pp_	Md	SD	0.7P,pp	T_0.7P,pp_	Md	SD	0.5P,pp	T_0.5P,pp_	Md	Sd
Matrix-AM	SP1	–	–	–	–	–	–	–	–	–	–	–	–
	SP2	–	–			–	–			–	–		
	SP3	–	–			–	–			–	–		
F1AM	SP1	3823	637	1022	450	2974	1174	1461	574	2124	1900	1984	754
	SP2	3248	1516			2526	2122			1805	2776		
	SP3	3896	912			3030	1086			2165	1276		
F3AM	SP1	8696	3452	3771	1673	6763	14893	11958	3427	4831	23960	19601	6148
	SP2	8522	5580			6628	12789			4735	22274		
	SP3	9042	2280			7033	8192			5024	12569		
F5AM	SP1	10904	10002	6339	3434	8481	18486	17256	4175	6058	34024	27969	8491
	SP2	8860	3193			6891	20678			4922	31620		
	SP3	11214	5821			8722	12605			6230	18263		
Matrix-HT	SP1	–	–	–	–	–	–	–	–	–		–	–
	SP2	–	–			–	–			–			
	SP3	–	–			–	–			–			
F1HT	SP1	6405	3889	2674	1327	4982	5203	3484	1636	3559	6877	4353	2297
	SP2	4891	2876			3804	3305			2717	3796		
	SP3	5210	1258			4052	1945			2895	2385		
F3HT	SP1	12920	7617	10572	5653	10049	11100	14747	7761	7178	15468	25726	10042
	SP2	14499	17090			11277	23660			8055	35537		
	SP3	10046	7010			7813	9481			5581	26174		
F5HT	SP1	22073	30989	29119	9185	17168	40273	39736	10418	12263	51374	52451	11346
	SP2	25385	37226			19744	49876			14103	64297		
	SP3	16935	19143			13172	29060			9409	41681		

Notes: P_max_ = P – maximum load or peak load; 0.9P,pp, 0.7P,pp and 0.5P,pp – points after the peak load, where the load is 0.9, 0.7 and 0.5 of the maximum load; T_0.9P,pp_, T_0.7P,pp_ and T_0.5P,pp_ – toughness at points 0.9P,pp, 0.7P,pp and 0.5P,pp; Md – average; Sd – standard deviation.

### 1.7 Pull-out response

**Fig. 5:**
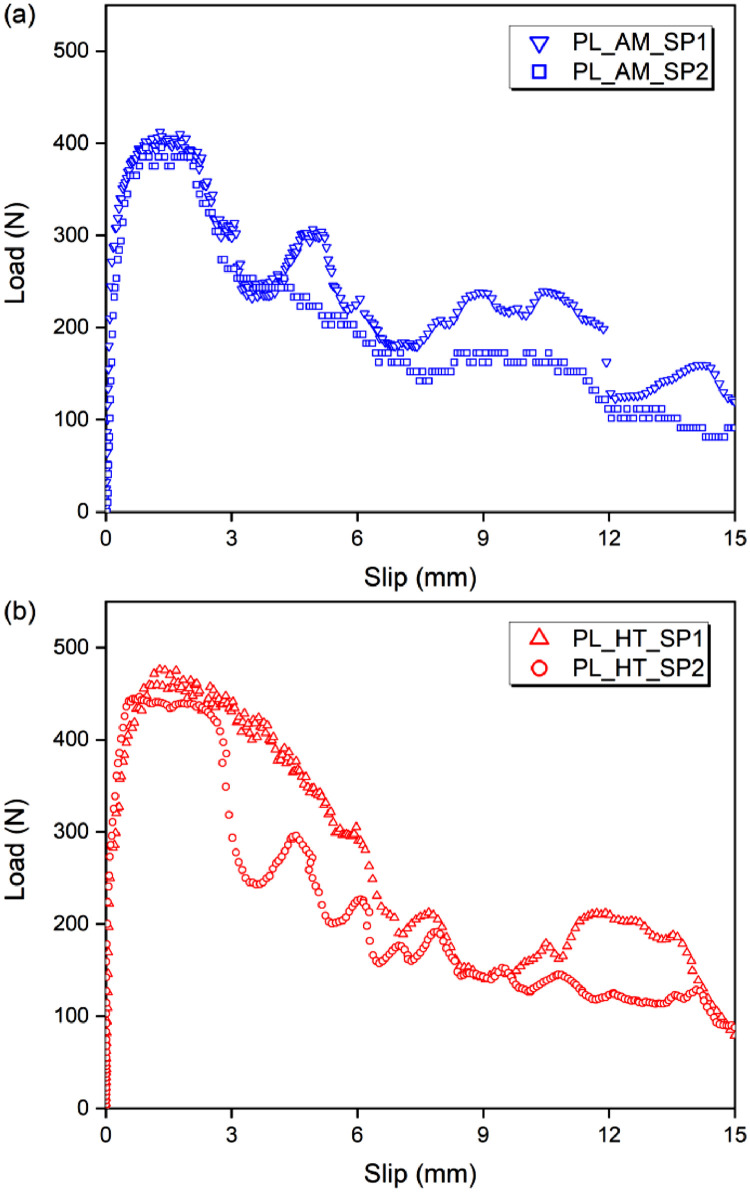
Load-slip curves of pull-out response (a) without heating and (b) after thermal treatment.

Fig. 5 shows the single fiber pull-out response. Two load-slip (P-s) curves are shown for each condition, each curve from one specimen. The blue data represents the AM condition and the red data the HT condition. PL means pull-out.

Table 16 shows the pull-out test parameters. The maximum load was between 412.75 N and 395.40 N without heating, and between 450.75 N and 479.01 N after heat treatment. The displacement in peak load was on average 1.2364 mm for AM and 1.3415 mm for HT. The pull-out energy was between 3140.53 N.mm and 4044.96 N.mm for AM, and between 3490.56 N.mm and 4293.77 N.mm after heating.

**Table 16:** Parameters of pull-out response.

Sample	P_max_ (N)	S_p,max_ (mm)	E_p_ (N*mm)	P_max_ (N)	Sd (N)	S_p,max_ (mm)	Sd (mm)	E_p_ (N*mm)	Sd (N*mm)
PL_AM CP1	405.59	1.5494	3140.53	404.58	8.718	1.2364	0.3390	3504.30	477.45
PL_AM CP2	412.75	1.2836	4044.96						
PL_AM CP3	395.40	0.8763	3327.43						
PL_HT CP1	460.74	1.7399	4059.21	463.5	14.330	1.3415	0.5735	3947.84	413.02
PL_HT CP2	479.01	1.6005	4293.77						
PLT_HT CP3	450.75	0.6842	3490.56						

Notes: P_max_ - maximum load; S_p,max_ - displacement at the point of maximum load; Ep - pull-out energy; Sd - standard deviation.

### 1.8 Fiber microstructure

**Fig. 6:**
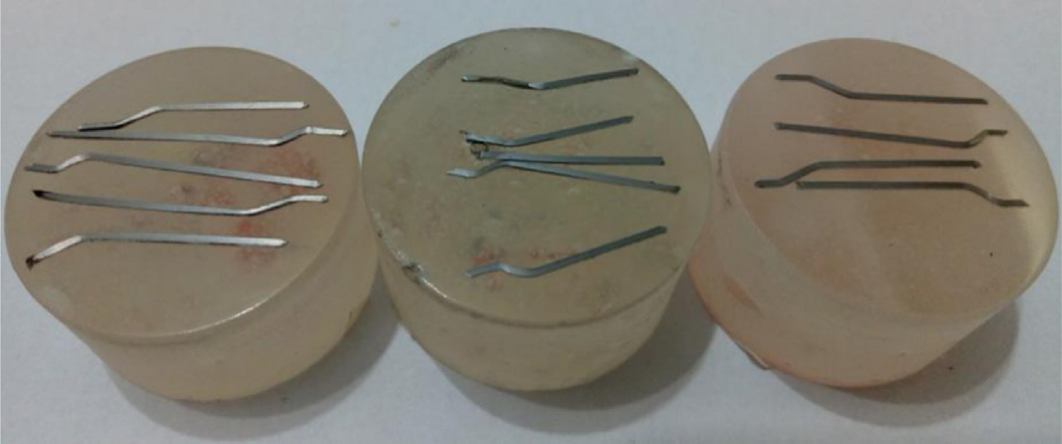
Samples for metallography and nanoindentation.

Fig. 6 shows the specimens for metallography and nanoindentation tests.

**Fig. 7:**
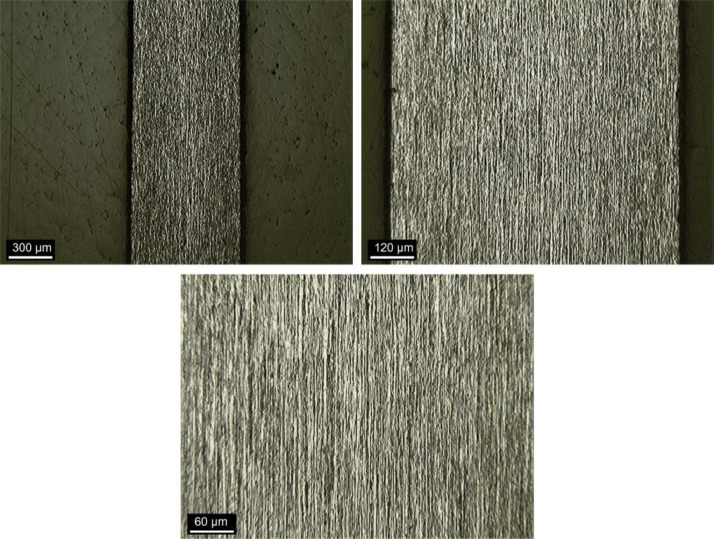
OM imagens of fibers “*as received*”.

**Fig. 8:**
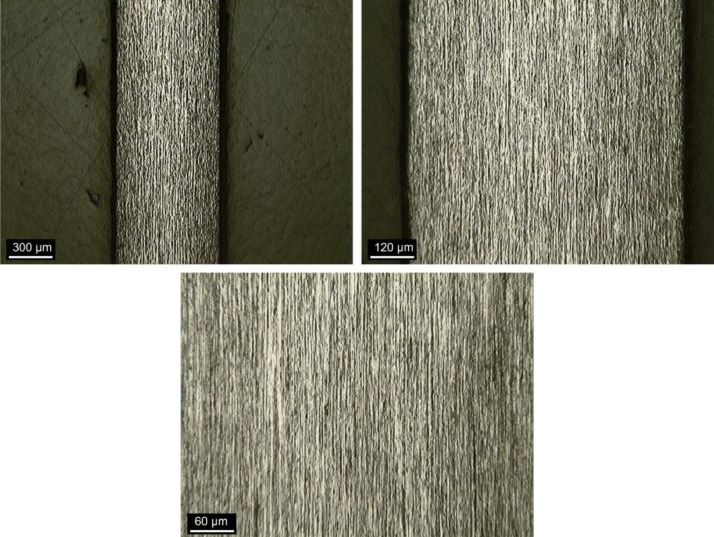
OM imagens of fibers after bending test.

**Fig. 9:**
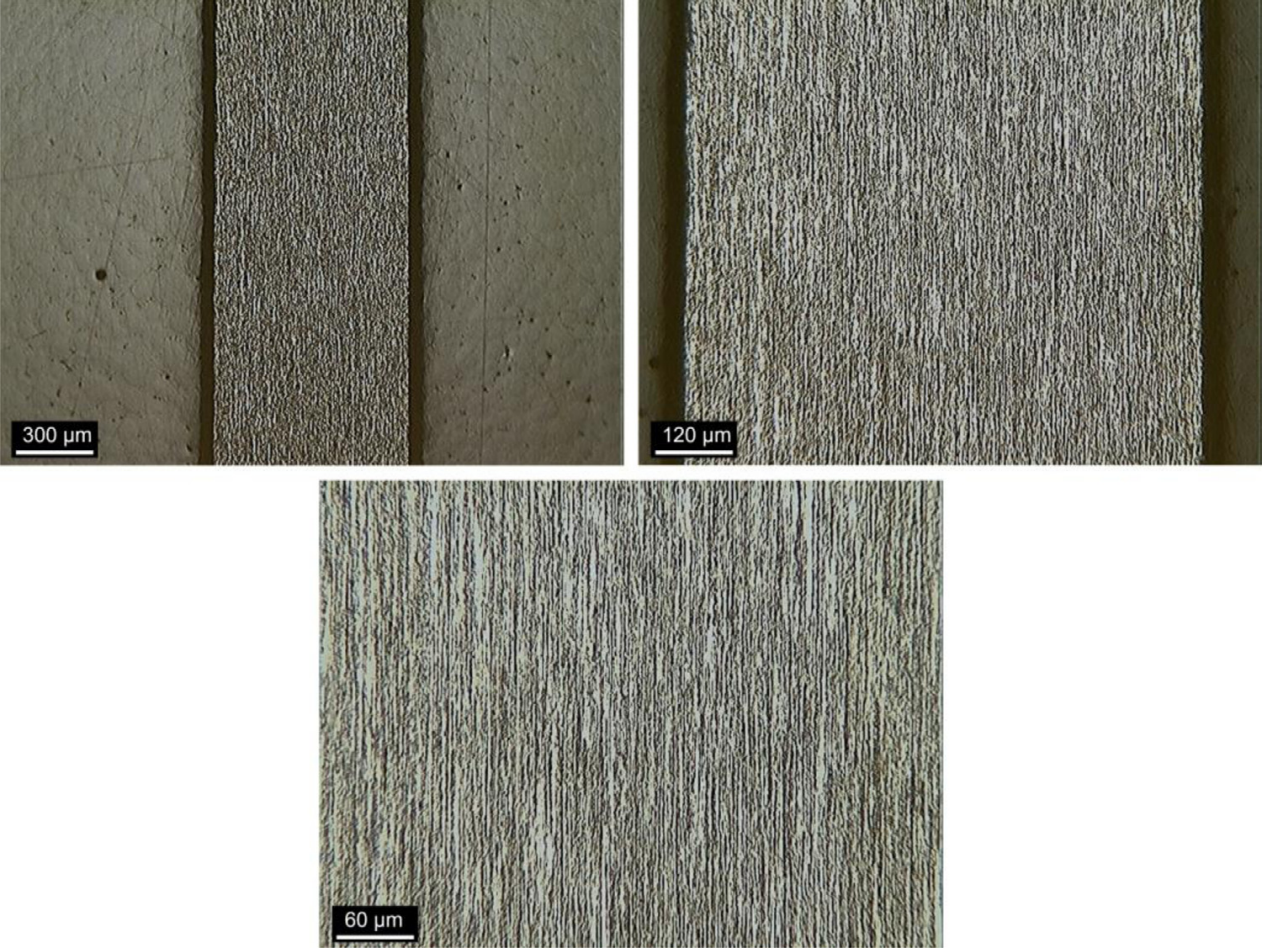
OM imagens of fibers after heat treatment.

Figs. 7, 8 and 9 show optical microscopy (OM) images of the steel fiber microstructure as received, after bending test and after high temperature submission, respectively.

### 1.9 Fiber hardness

**Fig. 10:**
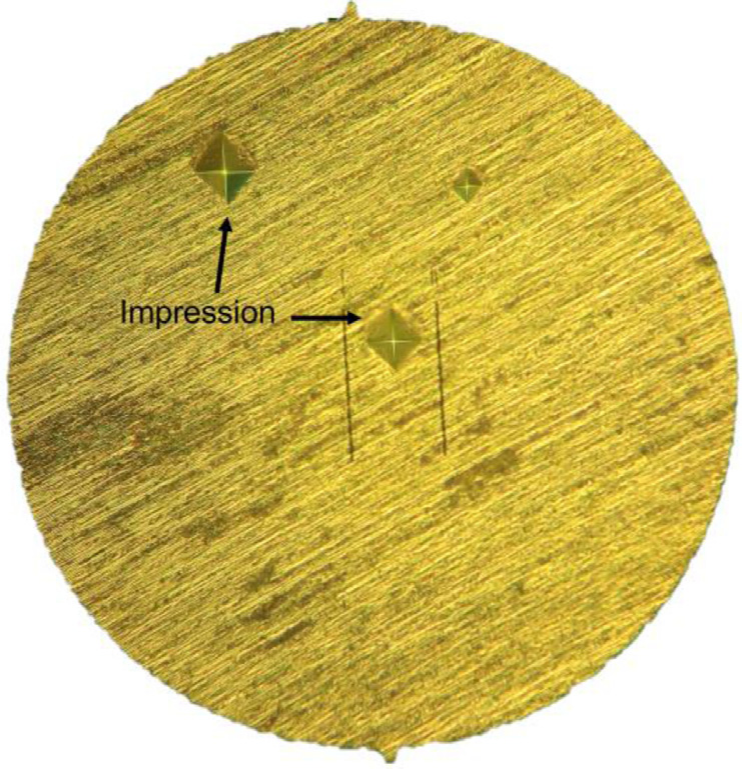
OM image during nanoindentation test.

Fig. 10 shows an image obtained by OM during the nanoindentation test, in which the typical microstructure of steel fibers and the impressions left by the indenter can be seen. Parallel bars were used as a reference.

Table 17 shows the hardness data obtained from six samples tested by nanoindentation. The fibers as received (AR) was an average hardness of 275 MPa. After flexural testing without heating, the average of hardness was 323 MPa. After submission to high temperature, the average of hardness was 277 MPa.

**Table 17:** Data of hardness measurements.

Measurements	Hardness Vickers (HV 0,2/15)		
	AR	AM	HT
1	286	327	267
2	294	342	285
3	264	337	283
4	272	313	279
5	266	302	271
6	284	318	279
Average	275	323	277
Standard deviation	11.04	13.78	6.37
Coefficient of variation	0.04	0.04	0.02

Notes: AR – as received; AM – reference condition; HT – high temperature.

**Fig. 11:**
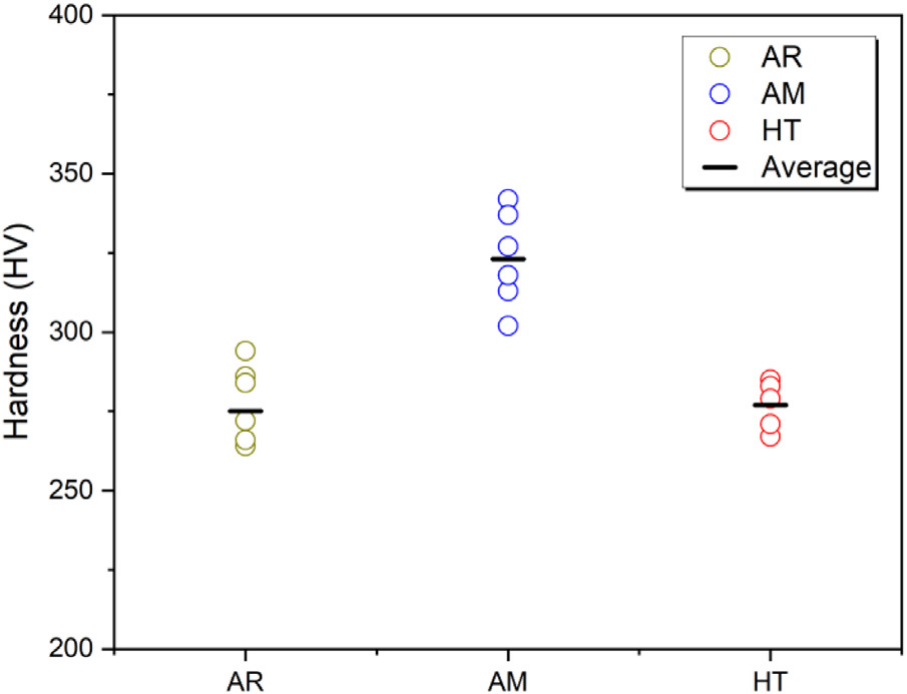
Data of hardness for AR, AM and HT conditions.

Fig. 11 graphically illustrates the hardness data for each condition mentioned.

### 1.10 Fiber surface

**Fig. 12:**
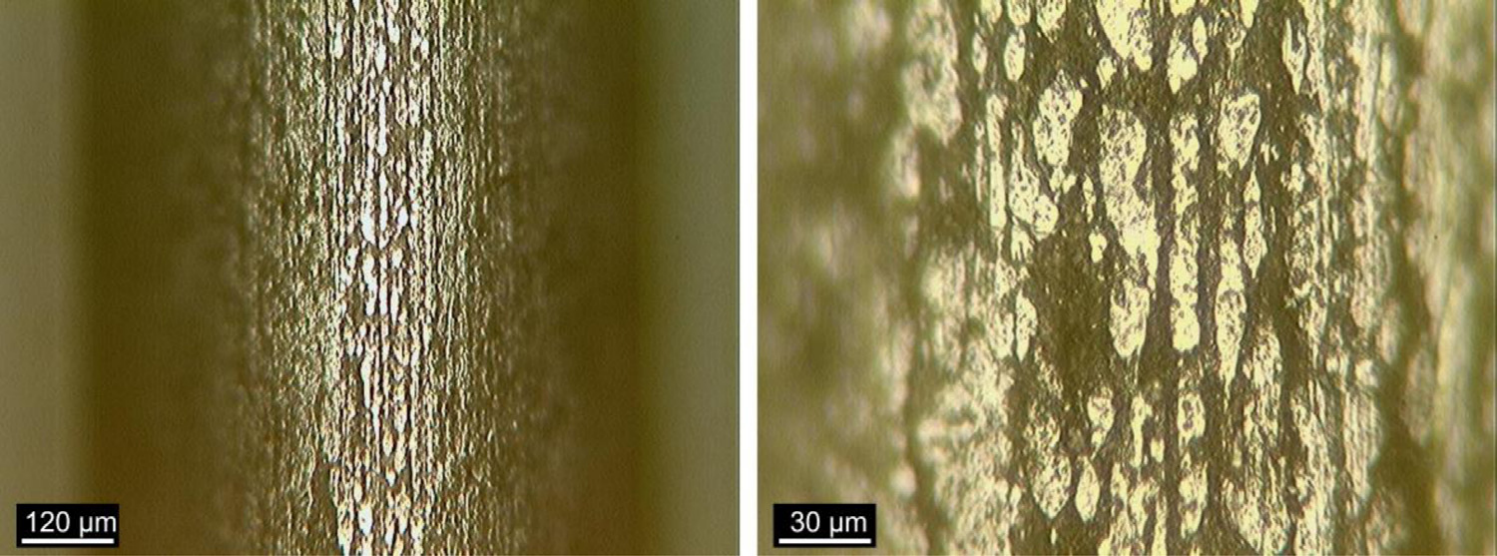
Fiber surface “*as received*” at (a) x100 and (b) x400 magnification. (Note: modified from [1]).

**Fig. 13:**
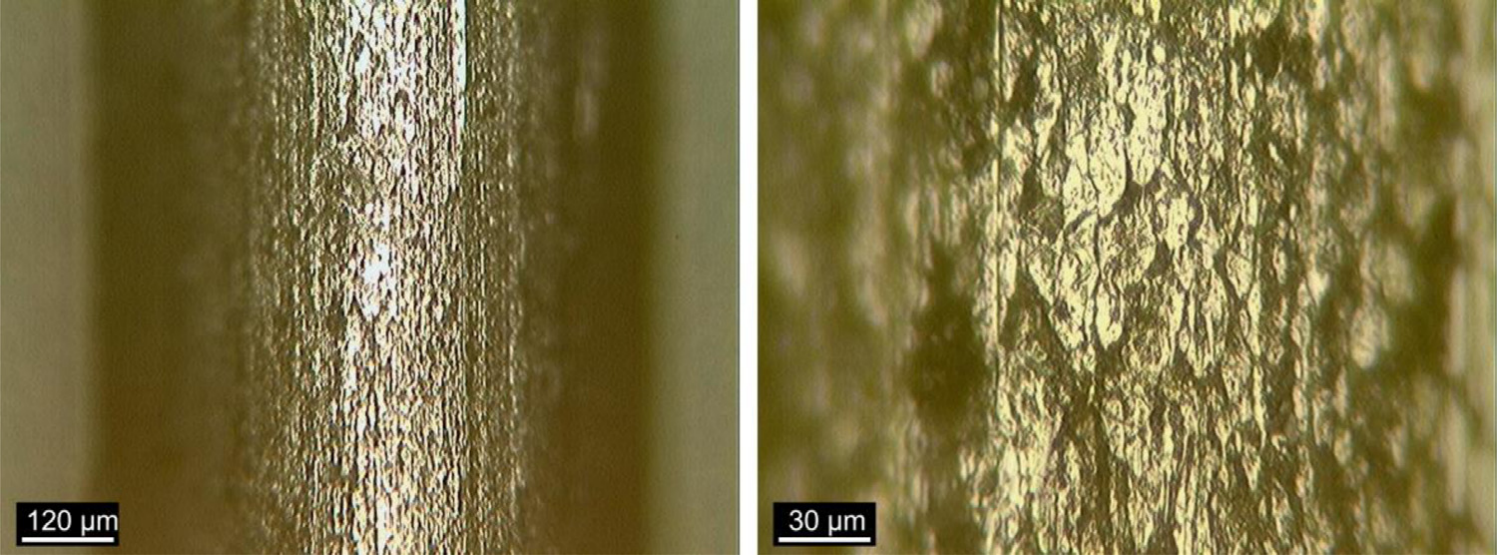
Fiber surface after bending test at (a) x100 and (b) x400 magnification. (Note: modified from [1]).

**Fig. 14:**
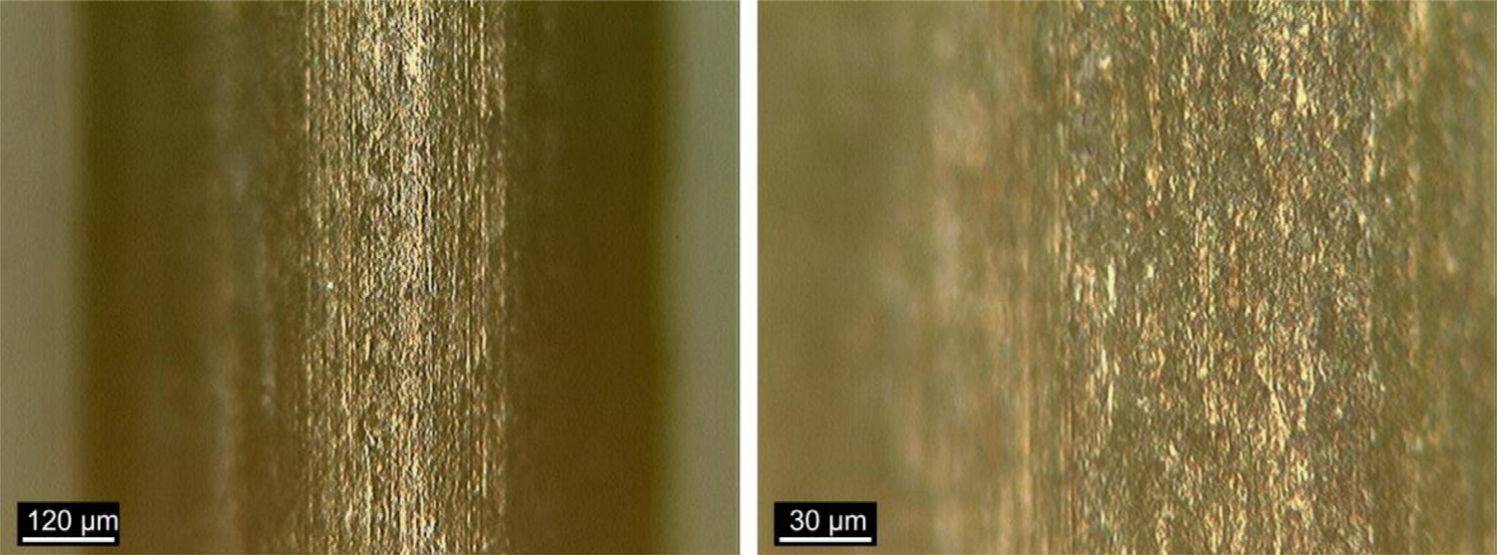
Fiber surface after heat treatment at (a) x100 and (b) x400 magnification. (Note: modified from [1]).

Figs. 12, Fig. 13, Fig. 14 show images obtained by OM of the fibers surface, as received, after bending test and after heat treatment, respectively. The x100 and x400 magnifications were used.

### 1.11 Fiber-matrix interface

**Fig. 15:**
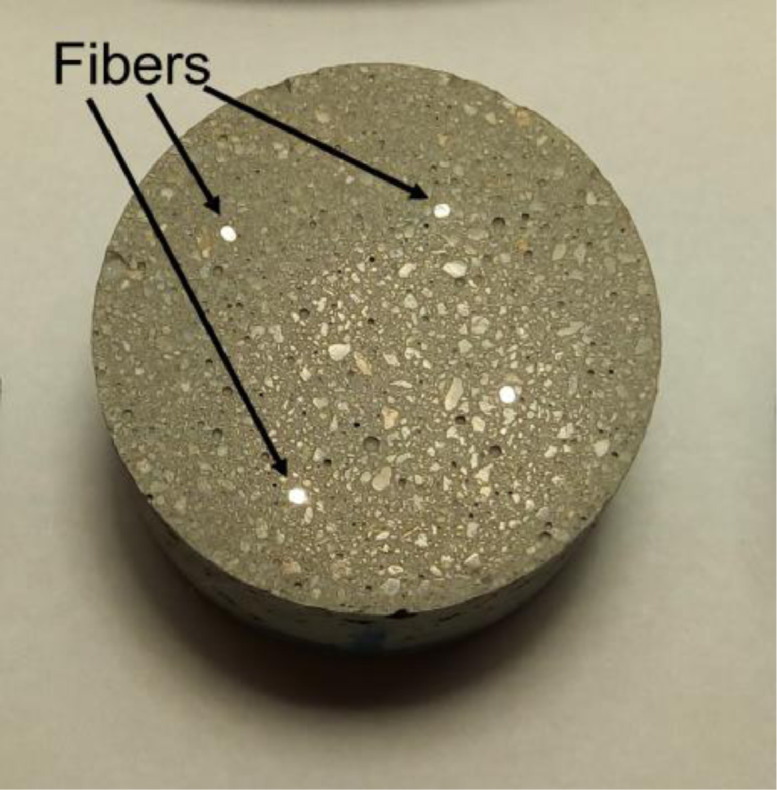
Sample for fiber-matrix interface analysis.

Fig. 15 shows a specimen prepared for fiber-matrix interface analysis by OM, after sanding and polishing. The fibers sections are the bright spots indicated.

**Fig. 16:**
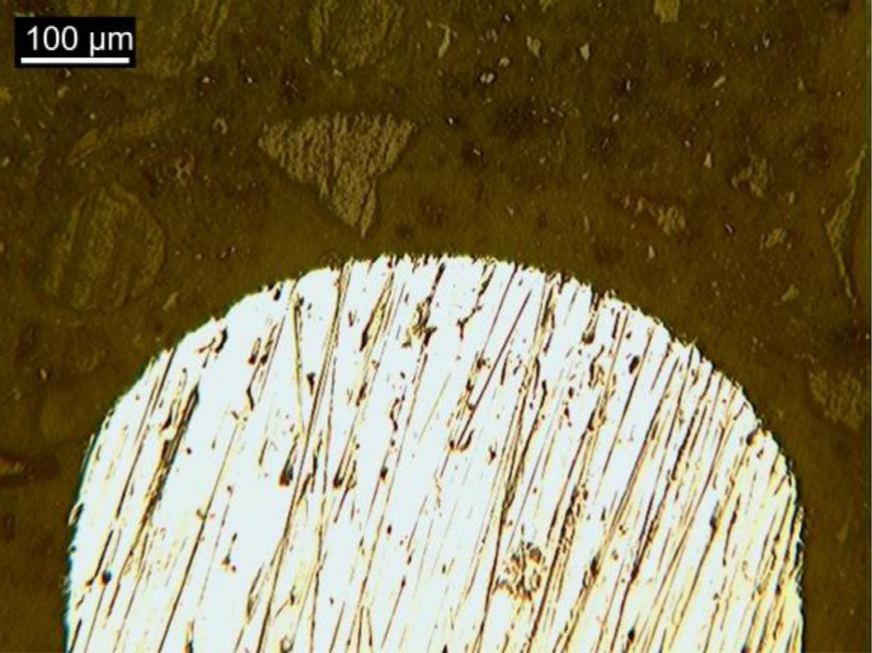
Fiber-matrix interface without heating at x100 magnification. (Note: modified from [1]).

**Fig. 17:**
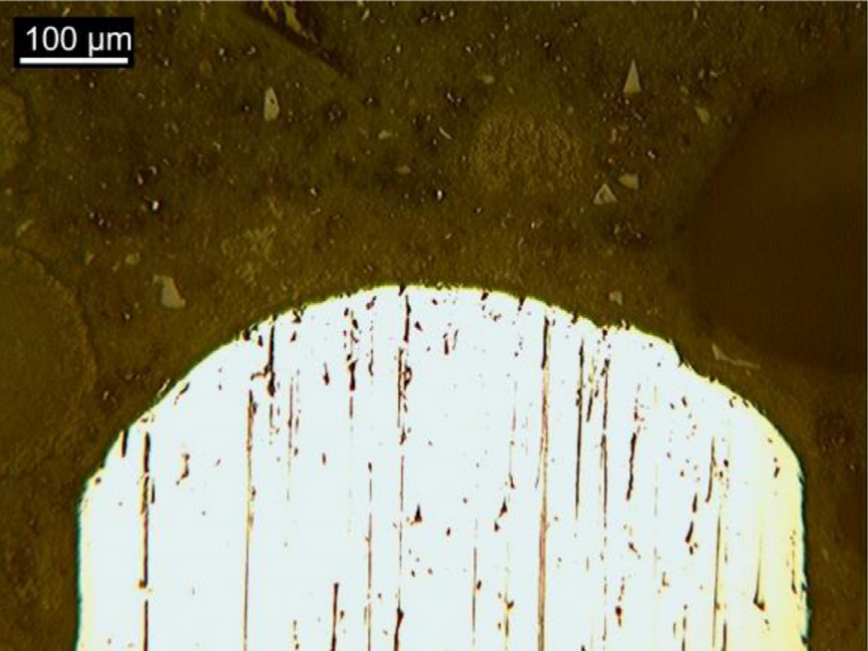
Fiber-matrix interface after heat treatment at x100 magnification. (Note: modified from [1]).

**Fig. 18:**
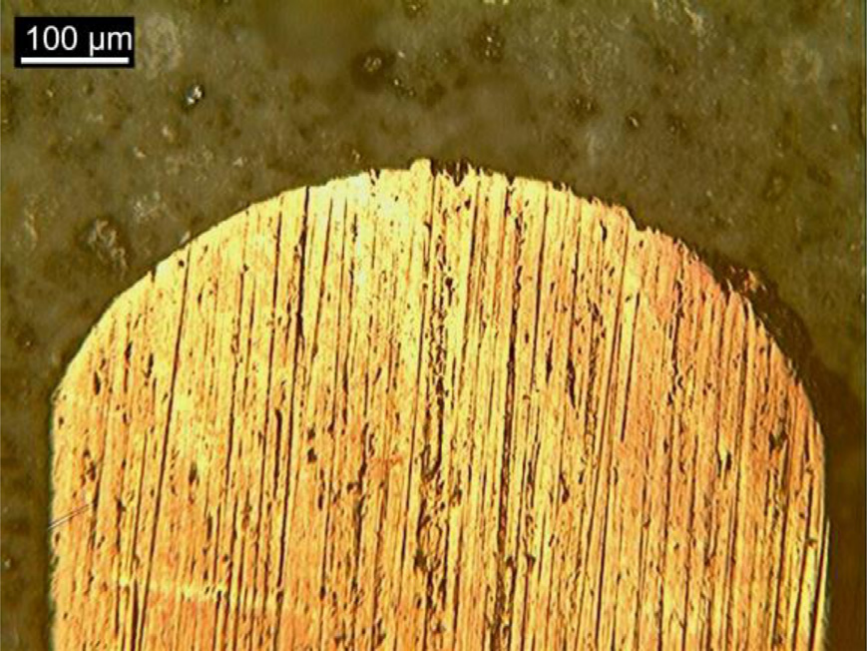
Fiber-matrix interface after heat treatment with previous preparation of the samples at x100 magnification. (Note: modified from [1]).

Figs. 16, 17 and 18 show images obtained by OM of the fiber-matrix interface, as received, after heat treatment, and after heat treatment with previous preparation of the samples, respectively. The x100 and x400 magnifications were used.

## 2 Experimental Design, Materials and Methods

The experimental phase was divided into three major stages. The first consisted in the selection and characterization of materials, as well as in the development of the cementitious composite. In the second stage, the specimens were produced. In the third stage, the specimens were tested to investigate the bending and compression behaviors, water absorption, porosity, density, fiber-matrix interface and surface, microstructure and hardness of the fibers. All tests were performed at the 7 days, before and after heat treatment. For additional information see related article in Ref. [1].

### 2.1 Molding of specimens

For molding the specimens, the slurry infiltrated fiber method was used, which consists of placing the fibers in the mold and then casting a fluid mortar, which fills the empty spaces between the fibers [4]. Prismatic mold with dimensions of 40 × 40 × 160 mm was used for bending. The fibers were placed in the molds according to the percentages of 1%, 3% and 5% by volume; finally casting a fluid mortar. The pull-out specimens were produced using cylindrical molds with dimensions of 50 × 100 mm. One of the hook-end of the fibers was sectioned to facilitate fitting into the test equipment. The other end of the fiber was embedded in the matrix. The length of the fibers embedded in the matrix was equal to 15 mm. The same cylindrical molds were used for compression and water absorption. The specimens for microstructural investigation and fiber characterization are described below.

After demolding within 24 h of mixing the materials, the specimens were placed in submerged curing in water saturated with calcium hydroxide, where they remained for seven days. After curing, the specimens were placed in an oven for a period of 24 h, at an average temperature of 65 °C, to remove excess water and carry out the tests. After the oven-drying, the specimens were removed and left at room temperature. Finally, half of the specimens were tested without heating, while the other half was subjected to high temperature and subsequent testing.

To produce cementitious grout, the literature suggests a water-cementitious materials ratio of 0.30; silica fume-cement ratio of 0.20; and sand-cementitious materials ratio of 1.23 [5]. Thus, the dosage was adjusted for the raw materials. The sand was dried in an oven for 24 h, at an approximate temperature of 105 °C, to completely remove the moisture. Then, the sieving process was adopted to obtain grains with a maximum dimension of 1.2 mm.

The variables proposed in this study were fibers content (1%, 3% and 5%) and thermal condition (with or without heat treatment). Thus, to differentiate the composites, a nomenclature was set up as follows: fiber percentage (F0, F1, F3 and F5 for 0%, 1%, 3% and 5% of fibers, respectively) and presence (HT) or absence (AM) of heat treatment.

### 2.2 Heat treatment

The heat treatment (HT) of the specimens was performed in an electric oven, to simulate a controlled condition for temperature variation with time. The temperature measurement was made using thermocouples attached to the specimens and the use of a glass wool thermal blanket. The other end of the thermocouple was connected to the digital meter, which converts the electrical signal into temperature values. To perform the heat treatment, the specimens were inserted in the oven along with sensors to measure the temperature, so that the heat flow occurred equally over the entire surface. Heating in the range of 200 °C to 300 °C promotes maintenance of properties. However, at higher temperatures the properties are deteriorated [6]. Around 400 °C, the propagation of micro-cracks in the matrix occurs, compromising the microstructure. At 560 °C, the decomposition of the structures of calcium silicate hydrate (C-S-H) occurs, porosity increases even more, and the microstructure is very cracked, which causes a rapid drop in strength. Thus, a temperature close to 260 °C was chosen to study the properties of FRCC in this specific condition and to evaluate the increment of residual strength.

### 2.3 Mechanical characterization of cementitious composite

The 4-point bending test was adopted to evaluate the mechanical behavior of FRCC, using a universal testing machine (UTM) from the manufacturer EMiC, located in the Department of Transports Engineering of CEFET/MG. The midpoint deflection was accurately measured by a deflectometer while the loading was measured by a load cell with a capacity of 300 kN. The resulting load and deflection values were captured and analyzed by TESC software, also from EMiC, generating a complete database. These data were collected and analyzed, resulting in the load-deflection (P-d) diagrams for each specimen. The bending stress were calculated using Eq. (1), considering the precepts of the bending test and considering the acting efforts, as follows:

(1) $$\sigma_{Flex}^{4P}=\frac{Pd}{bh^{2}}$$

Where $\sigma_{Flex}^{4P}$ is the 4-point bending stress, P is the load, d is the span (*d* = 132 mm), b is the width and h is the height (*b* = *h* = 40 mm).

### 2.4 Toughness

Toughness was calculated considering the area under the load-deflection curve (P-d). The ASTM C1609/C1609M [7] standard suggests the adoption of notable points to calculate the toughness when the deflections are equal to L/600 and L/150, where L is the distance between the supports. Kim, Naaman and El-Tawil [8] indicate the adoption of the other point (L/100) to fully characterize the behavior of FRCC using different fibers. In view of the conditions, the characteristics of the composite, the different fiber content and the extension of the P-d curves, it was necessary to adopt another notable point corresponding to the loads of 0.9P (before and after peak load), P (load of peak), 0.7P (post-peak) and 0.5P (post-peak). Therefore, the toughness points were T_0.9P,bp_, T_P,pc_, T_0.9P,pp_, T_0.7P,pp_ and T_0.5P,pp_.

### 2.5 Single fiber pull-out test

The present study adopted a specific setup for single fiber pull-out test, coupled to a universal testing equipment (See related article in Ref. [1]). A load cell with a capacity of 20 kN and accuracy of 0.001 N was used to measure the load. The specimen was attached by an adapter. A clamp was used to pull-out the fiber. To measure the fiber displacement accurately, an extensometer with accuracy of 0.001 mm was used. The adopted displacement rate was 10 µm/s, in agreement with Abdallah, Fan and Cashell [9].

### 2.6 Compressive strength of cementitious matrix

The mechanical strength of the cementitious matrix was evaluated by the axial compression test. Six cylindrical specimens were cast, half of which underwent heat treatment. All specimens were tested in compression using the universal testing equipment.

### 2.7 Fiber-matrix interface analysis

The fiber-matrix interface analysis was performed using a Kontrol optical microscope (MO), IM713. The fiber-matrix interface was studied using polished sections by MO to reveal the contour of the interface.

### 2.8 Characterization of steel fibers

The steel fibers were characterized under three different conditions. The first refers to the fiber as received, collected in the plastic container for conditioning materials. The second condition represents the fiber after the bending test, collected from the fractured specimen. The third condition represents the fiber subjected to heat treatment and after bending test. All fibers were chosen randomly, to obtain a reliable sample. Then, the fibers were divided in half with cutting pliers and inserted in silicone molds for cold mounting, which was done with transparent acrylic resin. The samples were prepared by sanding and polishing, then chemically attacked (Nital 3% reagent) to reveal the microstructure of the steel. The samples were taken under the Kontrol optical microscope, IM713, to obtain images of the microstructure of the steel. Finally, the samples were placed in the Shimadzu microdurometer, HMV, to evaluate the Vickers microhardness of the steel fibers. The microhardness test was performed with a load application of 1961 N (HV 0.2) and a holding time of 15 s.

## Ethics Statements

This work adheres to ethical publishing standards and does not include human studies, animal experiments or data collected from social media platforms.

## CRediT Author Statement

**Paulo Roberto Ribeiro Soares Junior:** Investigation; Data Curation; Writing – original draft preparation, Writing – review & editing; **Priscila de Souza Maciel:** Methodology, Writing – original draft preparation; **Elaine Carballo Siqueira Correa:** Methodology, Resources; **Augusto Cesar da Silva Bezerra:** Conceptualization, Supervision, Project administration, Funding acquisition.

## Declaration of Competing Interest

The authors declare that they have no known competing financial interests or personal relationships that could have appeared to influence the work reported in this paper.

## Data Availability

Raw data on fiber-reinforced cementitious composite subjected to high temperature (Original Data) (Mendeley Data) Analyzed data on fiber-reinforced cementitious composite subjected to high temperature (Original Data) (Mendeley Data).
